# Clinical Features Predicting COVID-19 Severity Risk at the Time of Hospitalization

**DOI:** 10.7759/cureus.57336

**Published:** 2024-03-31

**Authors:** Dikshant Sagar, Tanima Dwivedi, Anubha Gupta, Priya Aggarwal, Sushma Bhatnagar, Anant Mohan, Punit Kaur, Ritu Gupta

**Affiliations:** 1 Computer Science, Indraprastha Institute of Information Technology - Delhi, Delhi, IND; 2 Computer Science, Calfornia State University, Los Angeles, Los Angeles, USA; 3 Oncology, Dr. B.R.A Institute-Rotary Cancer Hospital, All India Institute of Medical Sciences, New Delhi, IND; 4 Centre of Excellence in Healthcare, Indraprastha Institute of Information Technology - Delhi, Delhi, IND; 5 Electronics and Communication Engineering, Indraprastha Institute of Information Technology - Delhi, Delhi, IND; 6 Onco-Anaesthesia and Palliative Medicine, Dr. B.R.A Institute-Rotary Cancer Hospital, All India Institute of Medical Sciences, New Delhi, IND; 7 Pulmonary, Critical Care and Sleep Medicine, All India Institute of Medical Sciences, New Delhi, IND; 8 Biophysics, All India Institute of Medical Sciences, New Delhi, IND

**Keywords:** imputation, feature selection, sars-cov-2, laboratory features, severity, artificial intelligence, machine learning, covid-19

## Abstract

The global spread of COVID-19 has led to significant mortality and morbidity worldwide. Early identification of COVID-19 patients who are at high risk of developing severe disease can help in improved patient management, care, and treatment, as well as in the effective allocation of hospital resources. The severity prediction at the time of hospitalization can be extremely helpful in deciding the treatment of COVID-19 patients. To this end, this study presents an interpretable artificial intelligence (AI) model, named COVID-19 severity predictor (CoSP) that predicts COVID-19 severity using the clinical features at the time of hospital admission. We utilized a dataset comprising 64 demographic and laboratory features of 7,416 confirmed COVID-19 patients that were collected at the time of hospital admission. The proposed hierarchical CoSP model performs four-class COVID severity risk prediction into asymptomatic, mild, moderate, and severe categories. CoSP yielded better performance with good interpretability, as observed via Shapley analysis on COVID severity prediction compared to the other popular ML methods, with an area under the received operating characteristic curve (AUC-ROC) of 0.95, an area under the precision-recall curve (AUPRC) of 0.91, and a weighted F1-score of 0.83. Out of 64 initial features, 19 features were inferred as predictive of the severity of COVID-19 disease by the CoSP model. Therefore, an AI model predicting COVID-19 severity may be helpful for early intervention, optimizing resource allocation, and guiding personalized treatments, potentially enabling healthcare professionals to save lives and allocate resources effectively in the fight against the pandemic.

## Introduction

The first confirmed case of coronavirus disease 2019 (COVID-19) in India was reported on January 27, 2020 [1]. Subsequently, it was realized that it is a highly contagious and moderately virulent virus that has spread worldwide in a short span of time, prompting the World Health Organization (WHO) to declare it as a pandemic on March 11, 2020. Countries worldwide faced three surges of COVID-19 cases in these past years, while the United States of America (USA) and India had some of the largest numbers of COVID-19. As of February 14, 2024, a total of 703.2 million confirmed cases of COVID-19 have been reported worldwide, accompanied by reported deaths exceeding 6.98 million [2]. Since COVID-19 created a huge personal, emotional, and financial loss at multiple levels, research on COVID-19 paced up rapidly in the recent past. Researchers invested themselves in different facets of this disease, including the modeling of trends of COVID-19 cases and the prediction of cases [3-5], diagnosing COVID-19 using chest X-rays and computed tomography scans [6,7], and studying heart-related disorders in post-COVID-19 patients [8,9].

So far, it has been observed that this disease with mild symptoms is self-limiting in the majority of patients [6]. However, in a considerable number of patients, COVID-19 infection can be severe and fatal [6]. Identification of such patients who are at a higher risk of developing severe disease or unfavorable outcomes at the earliest can be helpful for triaging, appropriate clinical decision-making, and resource reallocation. Severity prediction may also be helpful for doctors in prioritizing the treatment of patients who require immediate medical care.

Since the emergence of machine learning (ML)-based solutions specific to COVID-19 disease, a number of methods have been proposed in the literature for the identification and severity prediction of COVID-19 that may help medical practitioners in early COVID-19 treatment [4-24]. In this study, we propose a novel hierarchical artificial intelligence (AI) model, named hereby as COVID-19 severity predictor (CoSP), that is a multi-stage or hierarchical classification model for four-class COVID-19 severity prediction, developed on a large dataset of more than 7,000 patients, covering two waves of COVID-19 in India. We also address the problem of class imbalance in the dataset (i.e., of having an unequal number of patients in different severity groups/classes) while training the proposed model to alleviate the problem of overfitting to one class. We also carry out an interpretability analysis of the proposed CoSP model using SHapley Additive exPlanations (SHAP) [25]. There is generally no evidence of how accurately the features are extracted or learned by ML models because they are usually treated as black boxes. SHAP analysis helps us visualize importance scores or values, assigned to input features, that result in the predicted outcome. We evaluated the performance of the CoSP model on our large in-house collected dataset, which has a huge number of clinical and laboratory features. The CoSP model achieves improved performance compared to state-of-the-art ML methods, including random forest (RF), support vector machine (SVM), AdaBoost, gradient boosting, bagging, K-nearest neighbor (KNN), and neural network (NN). The salient points of this study are summarized below:

1. A hierarchical interpretable AI model, namely, CoSP, is developed that predicts COVID-19 disease severity at the time of admission to the hospital.

2. CoSP employs ML and utilizes simple clinical and laboratory features that can be made readily available without the need to wait for long. Prediction of disease severity at the time of hospital admission allows for optimizing decision-making and prioritization of the allocation of limited resources during COVID-19 wave peaks. Such a research methodology is also helpful in similar natural disasters.

3. Missing data values are imputed using a deep learning (DL)-based method, which can act as a potential solution for handling missing value problems in medical datasets.

4. A relatively small number of samples in some of the severity classes is a big challenge for training any ML model. The number of non-severe cases is much larger than severe cases, leading to a significant issue of class imbalance. This motivates us to tackle this problem of imbalanced data using class weighing and an ensemble learning method.

5. The proposed model’s learning and inference are interpreted using SHAP to understand the contributions of each laboratory parameter to the severity prediction.

6. A thorough comparison with state-of-the-art ML methods is carried out using the results validated on a large test dataset.

7. We have also built an interpretable CoSP calculator for COVID-19 severity prediction and made it publicly available (at http://covidseverity.sbilab.iiitd.edu.in/).

## Materials and methods

Dataset and preprocessing

**Figure 1:**
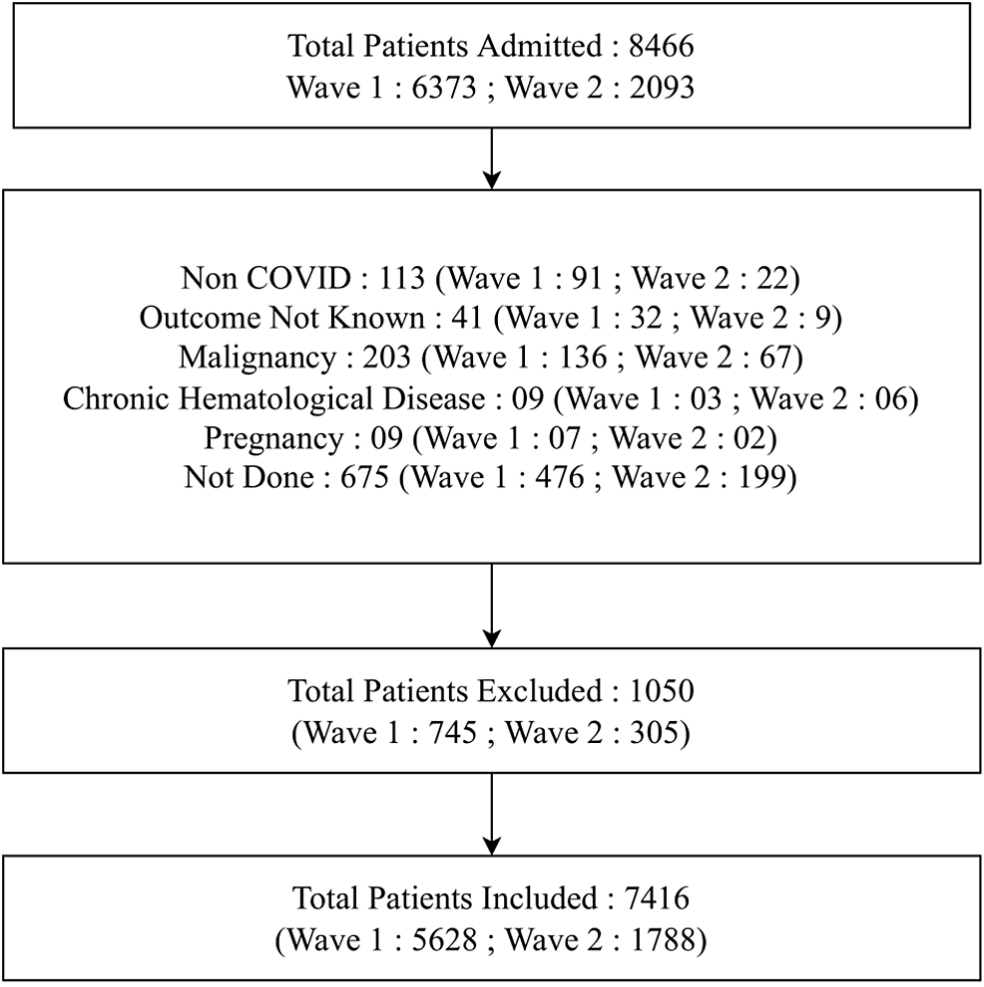
Flowchart depicting the enrollment of COVID-19 patients.

This retrospective study was conducted at the National Cancer Institute (NCI), All India Institute of Medical Sciences (AIIMS), Jhajjar. Ethical clearance of this study was taken from the institute's ethics committee (ref. no. IEC-373/08.05.2020). NCI-AIIMS is a specialized oncology institute that was converted into a designated COVID-19 treatment facility during the COVID-19 pandemic. All hospitalized patients were confirmed as COVID-19 cases by the laboratory tests of real-time reverse transcription polymerase chain reaction (RT-PCR) or cartridge-based nucleic acid amplification test (CBNAAT) or rapid antigen test (RAT). The dataset was gathered during the first wave (March 20, 2020, to January 20, 2021) and the second wave (April 9, 2021, to August 9, 2021) in India. Patients with non-confirmed COVID-19 laboratory reports were excluded from this study. Additionally, patients suffering from any malignancy or hematological diseases, as well as pregnant women patients, were not included in this study. Further, patients whose outcomes were not known, such as those transferred to another medical facility, or discharged on request (DOR), or left against medical advice (LAMA), were excluded from the study. Please refer to Figure 1 for more details.

Patients’ characteristics at the time of hospital admission were retrieved from the hospital information system. According to the clinical management guidelines on COVID-19 for symptomatic patients, issued by the Ministry of Health & Family Welfare (MoHFW), Government of India, on June 13, 2020, COVID-19 clinical severity was classified as mild, moderate, and severe at the time of hospital admission in the present study [26]. Patients with uncomplicated upper respiratory tract infection or non-specific symptoms, such as fever, cough, sore throat, nasal congestion, malaise, and headache, were classified to have mild disease. Patients with clinical features of dyspnea and/or hypoxia, fever, cough, SpO2 in the range of 90-93% on room air, and respiratory rate ≥ 24 per minute were classified as having moderate disease. Patients with pneumonia and fever were classified as having severe disease, plus one of the following: respiratory rate > 30 breaths/min, severe respiratory distress, and SpO2 < 90% on room air. The primary outcomes of this study were in-hospital mortality and the severity of the disease. The originally collected data consisted of 64 features, including useful demographic information (such as patient ID, date of admission, date of discharge, age, gender, COVID-19 waves (first or second), and clinical features such as the duration of stay in the hospital, severity of disease, clinical outcome), and 55 laboratory features that were collected as a part of baseline work-up at the time of admission in the hospital (Table 1).

**Table 1:** Details of the laboratory biomarkers evaluated in the study. (*) and italics indicate the features that were retained for building the proposed CoSP model.

Types of Features	Equipment Name	Company Name	Features Included [Units]
Hematological features	ADVIA 2120i hematology analyzer	Siemens AG, Germany	1. Hemoglobin (Hb) [g/dL]
			2. Hematocrit (HCT) [%]
			3. Red blood cell count (RBC) [10ˆ6/μL]
			4. Total leucocyte count (TLC) [10ˆ3/μL]
			*5. Platelet count (PLT) [10ˆ3/μL]
			6. Mean corpuscular volume (MCV) [fL]
			7. Mean corpuscular hemoglobin (MCH) [pg]
			8. Mean corpuscular hemoglobin concentration (MCHC) [g/dL]
			9. Red cell distribution width (RDW) [%]
			10. Neutrophils percentage [%]
			* 11. Absolute neutrophil count (ANC) [10ˆ3/μL]
			12. Lymphocytes percentage [%]
			*13. Absolute lymphocyte count (ALC) [10ˆ3/μL]
			14. Monocytes percentage [%]
			15. Absolute monocyte count (AMC) [10ˆ3/μL]
			16. Eosinophils percentage [%]
			*17. Absolute eosinophil count (AEC) [10ˆ3/μL]
			18. Basophils percentage [%]
			*19. Absolute basophil count (ABC) [10ˆ3/μL]
			*20. Neutrophil-to-lymphocyte Ratio (NLR; calculated by ANC by ALC)
			21. Platelet-to-lymphocyte ratio (PLR; calculated by PLT by ALC)
			*22. Lymphocyte-to-monocyte ratio (LMR; calculated by ALC by AMC)
			*23. Neutrophil-to-monocyte ratio (NMR; calculated by ANC by AMC)
			24. Systemic immune-inflammation index (SII; calculated by PLT × ANC/ALC) [ratio]
Biochemical Biomarkers	ADVIA 1800 biochemistry analyzer		25. Total bilirubin (TBIL) [mg/dL]
			26. Direct bilirubin (DBIL) [mg/dL]
			27. Indirect bilirubin (IBIL) [mg/dL]
			28. Alanine aminotransferase (ALT/SGPT) [U/L]
			*29. Aspartate aminotransferase (AST/SGOT) [U/L]
			30. Alkaline phosphatase (ALP) [I.U.]
			31. Total protein (TP) [g/dL]
			*32. Albumin (ALB) [g/dL]
			33. Globulin [g/dL]
			34. Albumin-globulin ratio (A/G) [ratio]
			*35. Urea [mg/dL]
			36. Creatinine [mg/dL]
			*37. Calcium [mg/dL]
			*38. Phosphorus [mg/dL]
			39. Uric acid [mg/dL]
			40. Sodium [mmol/L]
			41. Potassium [mmol/L]
			42. Chloride [mmol/L]
Inflammatory Biomarkers	Centaur XPT Immunoassay System and ADVIA 1800 biochemistry analyzer		*43. Ferritin [ng/mL]
			44. Procalcitonin [ng/mL]
			45. Interleukin -6 (IL-6) [pg/mL]
			*46. Lactate Dehydrogenase (LDH ) [U/L]
			47. C-reactive protein (CRP) [mg/dL]
			*48. CRP-albumin ratio (CAR) [ratio]
Coagulation Biomarkers	ACL TOP 750 LAS Hemostasis Testing System	Instrumentation Laboratory, USA	49. Prothrombin time (PT) [sec]
			*50. International normalized ratio (INR) [ratio]
			51. Activated partial thromboplastin time (APTT) [sec]
			*52. D-Dimer [ng/mL]
			*53. Fibrinogen [mg/dL]
Glycemic Biomarkers	ADVIA 1800 biochemistry analyzer	Siemens AG, Germany	54. Serum random glucose (Glu-R) [mg/dL]
			55. Glycated Haemoglobin (HbA1c) [%]

Data preparation

**Figure 2:**
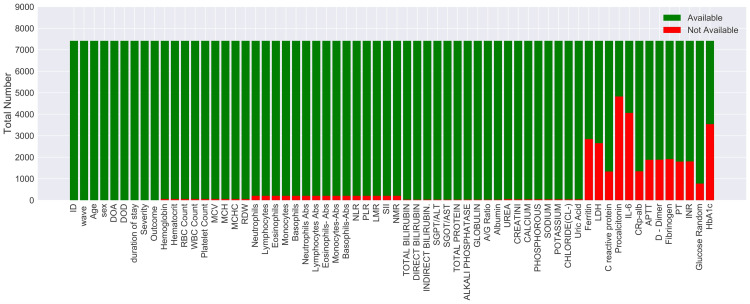
Bar plot showing the distribution of available versus missing values of each clinical parameter with reference to the total number of patients. DOA: Date of Admission, DOD: Date of Death, RBC: Red Blood Cell, WBC: White Blood Cell, MCV: Mean Corpuscular Volume, MCH: Mean Corpuscular Hemoglobin; MCHC, Mean Corpuscular Hemoglobin Concentration; RDW: Red cell Distribution Width, Abs: Absolute; NLR: Neutrophil-Lymphocyte Ratio, PLR: Platelet-to-Lymphocyte Ratio, LMR: Lymphocyte-to-Monocyte Ratio, SII: Systemic Immune-inflammation Index, NMR: Neutrophil-to-Monocyte Ratio, ALT/SGPT: Alanine Aminotransferase/Serum Glutamic Pyruvic Transaminase, AST/SGOT: Aspartate Aminotransferase/Serum Glutamic Oxaloacetic Transaminase, A/G Ratio: Albumin/Globulin, LDH: Lactate Dehydrogenase, IL-6: Interleukin-6, PT: Prothrombin Time, INR: International Normalized Ratio, HbA1c: Glycated Hemoglobin

The COVID-19 severity feature was used as the target-dependent variable with four unique values, namely, asymptomatic, mild, moderate, and severe, to develop a classifier that can classify the patients into the above four categories based on the given input features. However, there was a large class imbalance problem because the number of patients across categories differed on a large scale. Moreover, out of 7,416 patients, all 64 features were available for only 1,547 patients. Many features, such as ferritin, lactate dehydrogenase (LDH), C-reactive protein (CRP), procalcitonin, interleukin 6 (IL-6), C-reactive protein to albumin ratio (CAR), activated partial thromboplastin time (APTT), D-dimer, fibrinogen, prothrombin time (PT), international normalized ratio ( INR), serum glucose random (Glu-R), and glycated hemoglobin (HbA1c), were missing for a large number of patients, as shown in Figure 2.

Handling missing values

One approach to deal with the missing values is to drop all the samples that have any of the feature values missing. This would have resulted in a huge loss of data in our study because of several missing values across the entire cohort. Table 2 presents the distribution of the percentage of patients with missing feature values. From this table, we observe that all 64 feature values were available in 20.88% of patients, while the finally selected 19 features (the selection process is described in the next subsection on "Feature Selection") used to build the model were present in 45% of patients. In the dataset of 19 selected features, one to three features were missing in 32.81% of patients, while four or more features were missing in 22.08% of patients.

**Table 2:** Distribution of the percentage of patients in accordance with the number of missing features. %: percentage

Number of Missing Features	Patients (%)			
	Entire Dataset (64 Features)	Dataset with 19 Features		
		Entire Dataset	Train Set (80%)	Test Set (20%)
0	20.88	45.01	44.8	45.84
1	15.34	18.59	18.99	16.98
2	11.74	11.08	10.92	11.72
3	10.72	3.24	3.13	3.7
>=4	41.32	22.08	22.16	21.76

**Figure 3:**
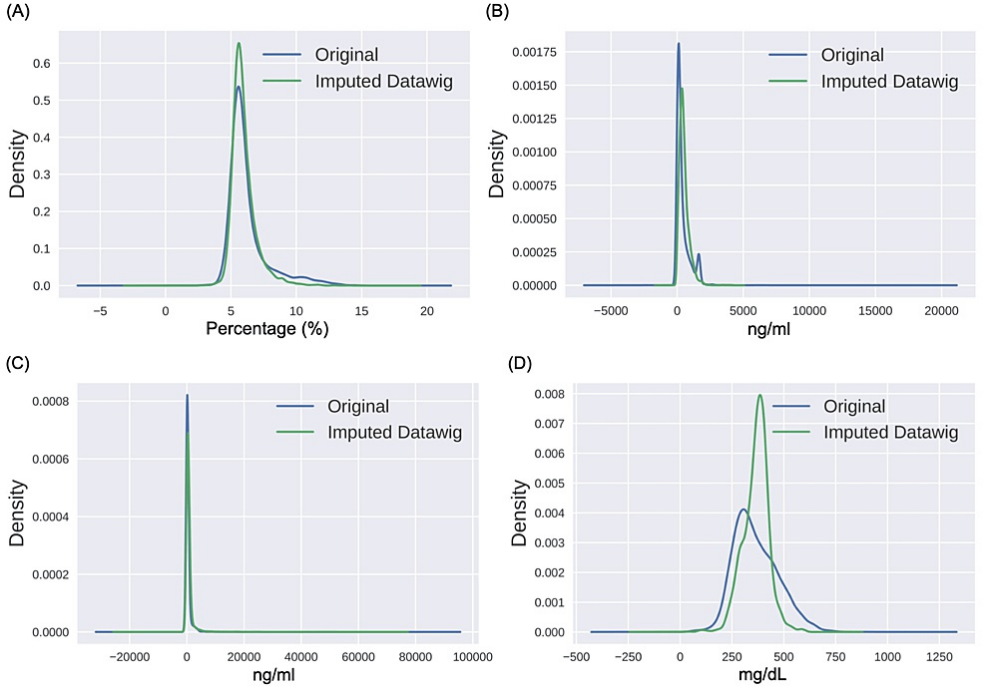
Density functions of the original feature data and the imputed data obtained from Datawig. Plots are shown for A) HbA1c (%), B) ferritin (ng/mL), C) D-dimer (ng/mL), and D) fibrinogen (mg/dL).

A similar distribution of patients was observed in the train and the test datasets, wherein approx. 45% of patients had all 19 feature values, while 22% of patients had four or more features missing. Thus, to handle a large number of missing feature values, we utilized a DL-based Python package called Datawig developed by Amazon [27] that helps in missing value imputation. This method considers features with missing values as dependent variables and features with all the values as independent variables. Further, the independent variables are used to fit a neural network-based classifier/regressor to the known values of the dependent variable, followed by predicting the missing values based on the fitted model. This method supports the imputation of both numerical and categorical variables. The performance of the imputation algorithm was assessed using the probability distribution (with and without imputation) on all features having missing values. For example, these distribution plots are shown for four features, namely, HbA1c, D-dimer, ferritin, and fibrinogen, in Figure 3. The probability distribution plot shows that the original distribution of values is quite close to the distribution of the imputed data on all four features. Similar observations were found in the rest of the features. Results were also verified using statistical methods. First, we performed the Wilcoxon rank-sum test and achieved a p-value >0.05 on all the features before and after the imputation, indicating that they had similar medians. Since the Wilcoxon rank-sum test compares only the medians, we also performed the Kolmogorov-Smirnov test with the null hypothesis that the probability distributions before and after the imputation were identical. We achieved a p-value >0.05 and, hence, failed to reject the null hypothesis, indicating that the distributions before and after were similar.

Handling class imbalance

We observed an unequal number of patients in all the four severity groups, i.e., 954 (12.86%) in asymptomatic, 4,295 (57.91%) in mild, 905 (12.20%) in moderate, and 1,262 (17.01%) in severe. This could lead to overfitting of the model to the majority class and, hence, could result in a biased model. In order to handle the class imbalance problem, we employed a weighted loss strategy while training. This training strategy assigns a higher weight to the training loss of the minority class and a lower weight to the majority class. In essence, more weightage is given to the minority class while training, which results in unbiased learning of the model.

Feature selection

**Figure 4:**
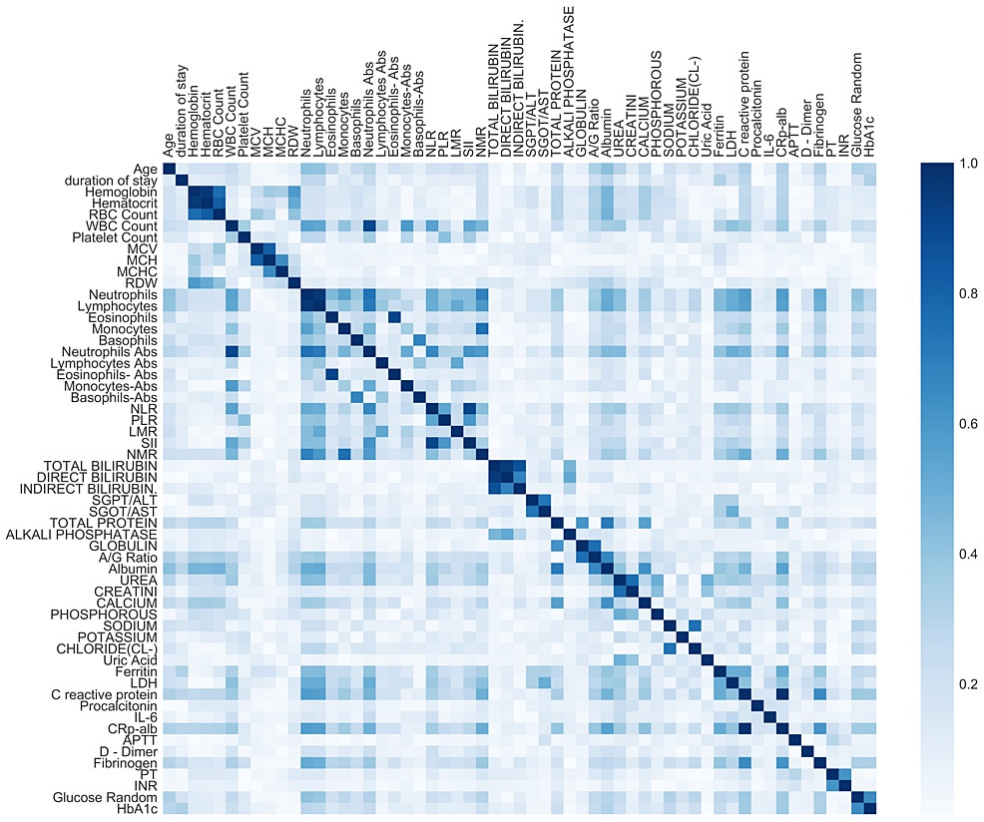
Heatmap showing Pearson correlation among the various features. RBC: Red Blood Cell, WBC: White Blood Cell, MCV: Mean Corpuscular Volume, MCH: Mean Corpuscular Hemoglobin; MCHC, Mean Corpuscular Hemoglobin Concentration; RDW: Red cell Distribution Width, Abs: Absolute; NLR: Neutrophil-Lymphocyte Ratio, PLR: Platelet-to-Lymphocyte Ratio, LMR: Lymphocyte-to-Monocyte Ratio, SII: Systemic Immune-inflammation Index, NMR: Neutrophil-to-Monocyte Ratio, ALT/SGPT: Alanine Aminotransferase/Serum Glutamic Pyruvic Transaminase, AST/SGOT: Aspartate Aminotransferase/Serum Glutamic Oxaloacetic Transaminase, A/G Ratio: Albumin/Globulin, LDH: Lactate Dehydrogenase, IL-6: Interleukin-6, PT: Prothrombin Time, INR: International Normalized Ratio, HbA1c: Glycated Hemoglobin.

Feature selection is an important step while training any classifier. It helps not only in reducing the dataset dimensionality but also in selecting the subset of features having high discriminative power. After going through an initial list of 64 features, we observed that a few features, such as COVID-19 wave, duration of hospital stay, outcome, and gender, were either insignificant for the classification task (because the variability of these features between the four severity classes was very low, and, hence, initial models trained on all 64 features provided us with very low importance values for these features) or could lead to the problem of target leakage in the COVID-19 severity classification task. Hence, we dropped these features from further data analysis. Next, we utilized Pearson correlation between features to drop one of the highly correlated features from the pairs of features. We realized that many features were highly correlated (Figure 4). A high absolute value of correlation indicates collinearity among features, which can also be defined as one feature implying another. Therefore, one of the two could be dropped for building the predictor. It is to be noted that the features were first normalized via min-max scaling in order to obtain standardization of data values before computing the correlation.

From Figure 4, we observe a high value of correlation among different features. The correlation matrix in this figure is a square matrix of size n × n, where n is the number of features in the dataset. The data value at the position of the i-th row and j-th column in this matrix denotes the correlation coefficient value between the i-th and j-th features. Values close to zero in this matrix indicate that the two features are independent of each other. A threshold value of 0.6 was selected in consultation with the doctors and also by running a number of experiments and, finally, selecting the one that yielded a consistent performance of the trained model. Below the correlation value of 0.6, we were losing informative features, and for a value greater than 0.6, the model was receiving a lot of redundant information, which was not improving the model’s performance. Thus, a threshold of 0.6 was finally selected for dropping one of the highly correlated features from the set of two correlated features. The selection of one feature from the pair of correlated features (with correlation coefficient >= 0.6) was made manually by the doctors based on the literature and medical domain knowledge, as outlined in Table 3. Further, APTT and procalcitonin features were dropped, as suggested in the literature [28]. Through this step, a total of 16 features were dropped, and we were now left with 41 features after this reduction.

**Table 3:** List of the features selected from the pair of two highly correlated features. RBC: Red Blood Cell, MCV: Mean Corpuscular Volume, MCH: Mean Corpuscular Hemoglobin; Abs: Absolute; NMR: Neutrophil-to-Monocyte Ratio, PLR: Platelet-to-Lymphocyte Ratio, SII: Systemic Immune-inflammation Index, CRP-Alb: C-Reactive Protein Albumin Ratio, PT: Prothrombin Time, INR: International Normalized Ratio, HbA1c: Glycated Hemoglobin

Feature Pair	Correlation	Dropped	Reason
Hematocrit - Hemoglobin	0.9632	Hematocrit	We evaluated the hemoglobin and red blood cell indices because anemia is known to be associated with poor recovery from illness. The hemoglobin and MCV were retained as they were measured directly [28].
RBC Count - Hemoglobin	0.7627	RBC Count	
MCH - MCV	0.8164	MCH	
Neutrophils Abs - WBC Count	0.9067	WBC Count	Absolute differential white blood cell count was included instead of relative counts, and the calculated parameters were dropped [28].
NMR - Neutrophils	0.6804	Neutrophils	
Neutrophils Abs - Lymphocytes	0.7137	Lymphocytes	
Eosinophils- Abs - Eosinophils	0.9174	Eosinophils	
Basophils-Abs - Basophils	0.6726	Basophils	
SII - Neutrophils Abs	0.6084	SII	
SII - PLR	0.6002	SII and PLR	
DIRECT BILIRUBIN - TOTAL BILIRUBIN	0.9521	DIRECT BILIRUBIN	As per literature [29]
CRP-Alb - C reactive protein	0.9851	C Reactive Protein	For CRP-Alb ratio calculation, both C-reactive protein (CRP) and albumin are used, and therefore, CRP was excluded [30].
INR - PT	0.6135	PT	PT is used to calculate INR; hence, it was excluded [31].
HbA1c - Glucose Random	0.6435	Glucose Random	Diabetes may influence recovery from infectious diseases, and thus, HbA1c, which is a better indicator of glycaemic control in diabetics, was evaluated.

In the second feature elimination, reduction in features was carried out using the Wilcoxon rank-sum test done between asymptomatic vs. mild and moderate vs. severe patients for every feature as these subgroups had the most overlap in the feature space. We dropped the features with p-value >0.05 across asymptomatic vs. mild as well as across moderate vs. severe groups of patients. This process resulted in a total of 19 features for all the 7,416 patients finalized for building the model. After this data preparation, we randomly selected 80% of the data for model training and 20% for model testing. The train-test splits were done in a random stratified manner, where care was taken to maintain similar percentages of different classes in the train and test splits.

Model development

**Figure 5:**
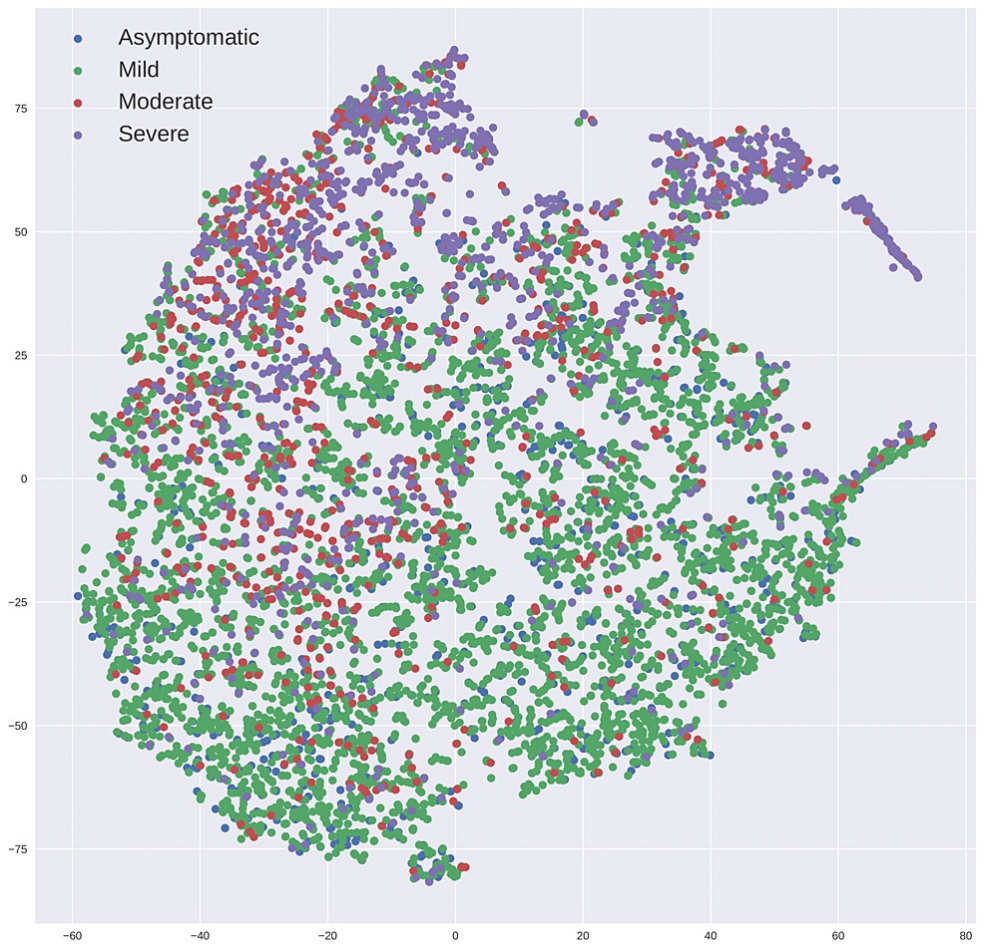
t-SNE plot showing the patient samples (using their features) projected to a 2D Euclidean space. Patients of different COVID-19 severity classes are shown with different colors. This graph allows us to view the separation of patients belonging to different classes.

Once the significant features are selected, they are also visualized using the t-distributed symmetric neighbor embedding (t-SNE) plot (Figure 5). t-SNE is a nonlinear dimension reduction technique that projects high-dimensional data into low-dimensional space to visualize their similarities and separation. The visualization plot from Figure 5 reveals that there is a large overlap among the four classes. The highest overlap is observed among the samples of asymptomatic and mild classes. Samples of moderate and severe classes also display considerable overlap. The model is built using 19 features, i.e., each subject’s sample exists in a 19-dimensional Euclidean feature space. Figure 5 is a projection of the dataset of 19-dimensional space to a two-dimensional space using a dimensionality reduction algorithm called t-SNE for the visualization of the dataset to provide an essence of how the data exist in a higher dimensional plane. Since this dataset has four classes, Figure 5 shows the extent of overlap of the data points of four classes, depicting the high complexity of the problem because it is difficult to distinguish between asymptomatic and mild patients of COVID-19 medically as well. This difficulty translates to difficulty in finding a hypothetical complex nonlinear decision boundary that can distinguish these four classes with our ML model.

**Figure 6:**
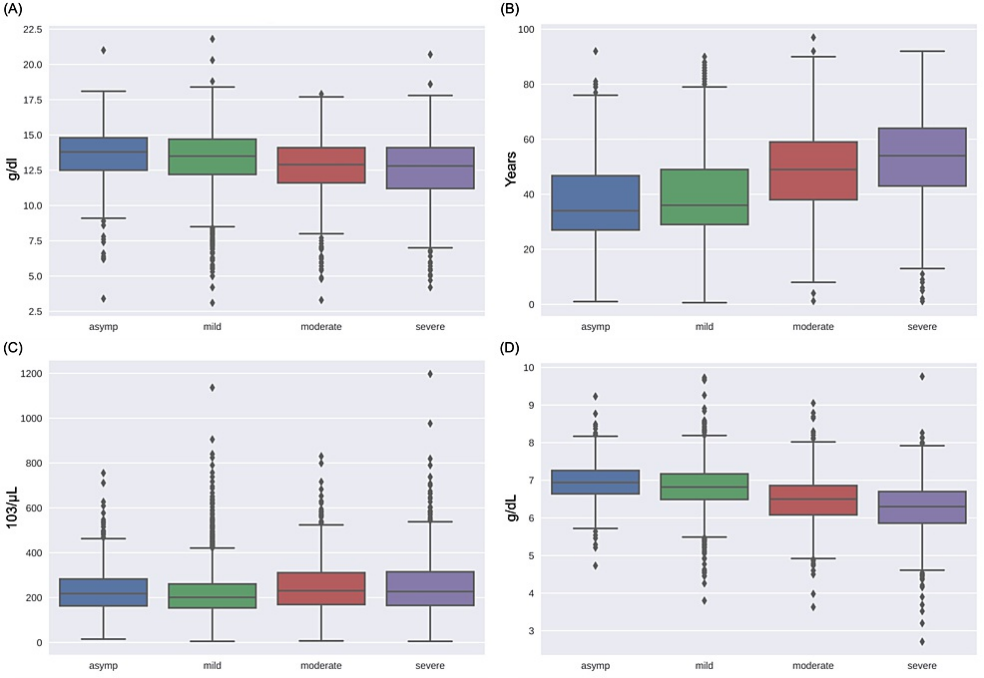
Class-wise feature distribution using box plots for A) hemoglobin (g/dL), B) age (years), C) platelet count (103/μL), and D) total protein (g/dL).

The underlying topological similarities or differences among various clinical and laboratory features of patients among the four severity groups are also visualized using boxplots in Figure 6. We observed an increasing median value trend of age with the increase in the patient’s severity, while a decreasing trend is observed in hemoglobin and total protein as the severity increases. No significant trend was observed for the platelet count feature. Moreover, interquartile ranges of all these features showed significant overlap among all the four risk groups. This indicated a low discriminative power among various features of all the four severity groups. 

**Figure 7:**
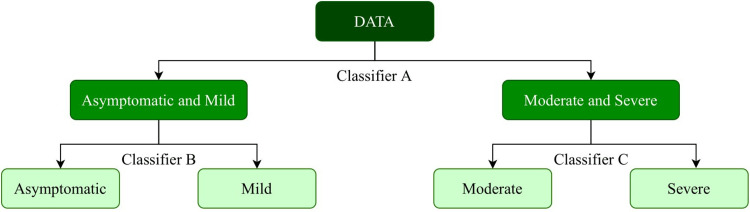
Hierarchical training strategy employed to build the CoSP model.

This motivated us to propose a multi-stage classification strategy in this paper (see Figure 7), where we first classified the patients into two groups, namely, group 1 (asymptomatic, mild) and group 2 (moderate, severe). Further, two more classifiers are trained on both groups individually. We utilized multiple classifiers and selected the random forest classifier for all the classifier stages because it outperformed other classifiers for robust learning of all four classes. Random forest is capable of handling class imbalance and high collinearity between features, which proved to be an excellent candidate for the classifiers in our multi-model approach.

Comparison of Performance with Other Standard ML Algorithms

We trained several popular ML algorithms for the proposed four-class classification for evaluating and comparing our proposed approach. It includes training RF, SVM, AdaBoost, gradient boosting, bagging, KNN, and NN models. These are some well-known algorithms used for the classification tasks for COVID-19 datasets. A brief description of each of these algorithm is presented in the supplementary material.

Performance Evaluation Matrices for Machine Learning Models

We evaluated the performances of the models using the area under the curve for the receiver-operating characteristic (AUC-ROC) and weighed F1 score and accuracy. The AUC-ROC curve is an excellent metric to assess classification performance and is useful, in particular, when there is a class imbalance problem. It is a performance measurement for classification problems at various threshold settings evaluated according to their sensitivity, specificity, positive predictive value, and negative predictive value. Here, ROC is a probability curve, and AUC captures the degree of separability between the classes. Its value ranges between 0 and 1. A higher value of AUC-ROC indicates that the model is capable of good prediction of classes. 

**Figure 8:**
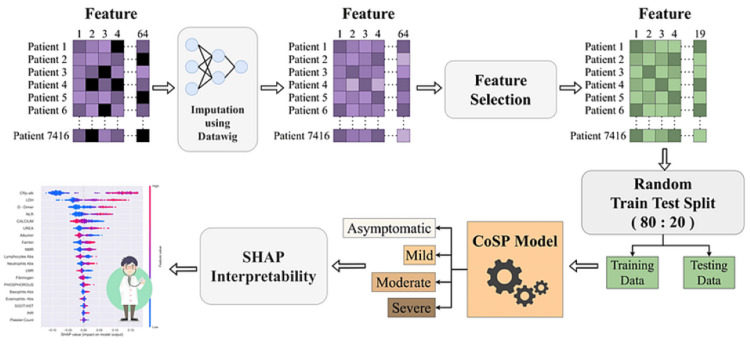
Complete workflow of the development of the proposed CoSP methodology.

The complete workflow of the development of the proposed CoSP methodology is depicted in Figure 8.

## Results

Clinical and laboratory characteristics of COVID-19 patients

A total of 7,416 patients were included in this study, as shown in Figure 1. The median age of the patients was 40 years (range: 01-97 years). The majority of the patients were males (5,485; 73.96%), followed by females (1,927; 25.98%) and others (4; 0.05%). At the time of admission, 4,295 (57.92%) had mild disease. Meanwhile, 1,262 (17.02%) had severe disease, and 954 (12.86%) were asymptomatic, followed by patients with moderate disease (905; 12.20%). Regarding disease outcome, 461 (6.22%) expired, and 6,955 (93.78%) recovered from the illness. The average duration of the hospital stay was 10 days (range: 3-22 days).

Training details

The code to develop the models was written in Python using open-source libraries such as scikit-learn, tensorflow, etc., for simulating multiple classifiers. Optimal hyperparameters for these classifiers were found using a fivefold cross-validation-based Grid Search. For the neural network, the optimal hyperparameters (number of neurons, layers, learning rate, etc.) were found using an open-source tool called Keras tuner.

Results on the identification of moderate/severe COVID patients

Most methods in the literature have carried out binary classification for the identification of moderately severe/severe class from the asymptomatic/mild COVID class, as evident from Table 4.

**Table 4:** Literature review on COVID severity prediction. CAR: C-Reactive Protein/Albumin Ratio, LDH: Lactate Dehydrogenase, NLR: Neutrophil-Lymphocyte Ratio, NMR: Neutrophil-to-Monocyte Ratio, ALC: Absolute Lymphocyte Counts, CRP: C-Reactive Protein, IL-6: Interleukin-6, DBIL: Direct Bilirubin, TBIL: Total Bilirubin, ALT/SGPT, Alanine Aminotransferase/Serum Glutamic Pyruvic Transaminase, AST/SGOT, Aspartate Aminotransferase/Serum Glutamic Oxaloacetic Transaminase, APTT: Activated Partial Thromboplastin Time, CK: Creatinine Kinase, cTnI: Troponin I, cTnT: Troponin T, hs: High Sensitivity, RDW: Red Cell Distribution Width, BUN: Blood Urea Nitrogen, SOFA Score: Sequential Organ Failure Assessment Score, RF: Random Forest, SVM: Support Vector Machines, UMAP: Uniform Manifold Approximation and Projection, KNN: K-Nearest Neighbour, GBM: Gradient Boosting Machines, DL: Deep Learning, NN: Neural Networks, LR: Logistic Regression, DT: Decision Tree

Study	Outcome	Determinant Features	ML Approach	Samples	Classes
Present Study	Prediction of severity	CAR, LDH, D-Dimer, NLR, ferritin, albumin, urea, NMR, calcium, and ALC	RF-based hierarchical classification approach	7416	4
Yao et al. [11]	Prediction of severity	Age, neutrophil %, calcium, monocyte %, urine test values (urine protein, red blood cells (occult), and pH (urine))	SVM	137	2
Laatifi et al. [12]	Prediction of severity	CRP, platelets, and D-dimers	UMAP, AdaBoost, Extreme Gradient Boosting, RF	337	2
Navlakha et al. [13]	Prediction of severity	Ferritin and IL 6	RF	348	3
Aktar et al. [14]	Prediction of severity	Respiratory rate, lactate, blood pressure, hemoglobin, hematocrit, venous and arterial base excess, neutrophils, albumin, urea, platelet count, and potassium	SVM, KNN, GBM, DL	545	2
Statsenko et al. [15]	Prediction of severity	Lymphocyte count, TBIL, AST, ALT, D-dimer, APTT, CK, CRP, LDH, troponin, ferritin, fibrinogen	NN, AdaBoost, Gradient Boosting, RF	560	2
Zhao et al. [16]	Prediction of severity	IL-6, cTnI, procalcitonin, hs- CRP, chest distress, calcium	SVM	172	2
Huang et al. [17]	Prediction of severity	Comorbidities, respiratory rate, CRP, LDH	LR	125	2
Zhou et al. [18]	Prediction of severity	Age, CRP, D-dimer, product of N/ L*CRP**D-dimer	LR	377	2
Zhu et al. [19]	Prediction of severity	IL-6, CRP, hypertension	LR	127	2
Han et al. [20]	Prediction of severity	LDH, lymphocyte counts, AST, CPR, SOFA score, CT score	LR	47	2
Aloisio et al. [21]	Prediction of severity	Age, cTnT, LDH, CRP, albumin, D-dimer, ferritin	LR	427	2
Gong et al. [22]	Prediction of severity	Older age, higher LDH, CRP, RDW, BUN, and direct bilirubin, lower albumin	LASSO regression, DT, RF, and SVM, and LR	372	2
Terwangne et al. [23]	Prediction of severity	WHO severity classification, acute kidney injury, age, LDH, lymphocytes, APTT	Bayesian network analysis	295	3
Liang et al. [24]	Prediction of severity	Age, hemoptysis, unconsciousness, comorbidities, cancer history, neutrophil-to-lymphocyte ratio, LDH, DBIL	LASSO then LR	2300	2

Hence, first, we present the results of the proposed classifier A (Figure 7) that carries binary classification for the identification of moderate/severe class patients from those belonging to asymptomatic/mild COVID class patients. Table 5 presents the results of this binary classification problem obtained using the CoSP-Classifier-A vis-a-vis those quoted in the literature on AUC-ROC, accuracy, and weighted F1 score.

**Table 5:** Results on the identification of moderate/severe COVID patients (binary classification). AUC-ROC: Area under the receiver operating characteristic curve.

Model	Total Sample Size	Test Sample Size	AUC-ROC	Accuracy (%)	Weighted F1-Score
Yao et al. [11]	137	27	-	81.48	-
Laatifi et al. [12]	337	101	1.00	-	-
Aktar et al. [14]	545	109	0.90	-	0.94
Statsenko et al. [15]	560	56	0.86	-	-
Zhao et al. [16]	172	34	-	91.38	-
Huang et al. [17]	125	-	0.94	-	-
Zhou et al. [18]	377	113	0.88	-	-
Zhu et al. [19]	127	-	0.90	-	-
Han et al. [20]	47	-	0.98	-	-
Gong et al. [22]	372	165	0.85	-	-
Liang et al. [24]	2300	710	0.88	-	-
CoSP-Classifier-A	7416	1484	0.91	86.65	0.87

Results of the proposed four-class CoSP model

Next, we built the four-class CoSP model using the hierarchical modeling approach shown in Figure 7 for the identification of asymptomatic, mild, moderate, and severe classes. Due to the lack of availability of datasets used in previous studies or a large benchmark dataset for this problem, we trained and tested various models used by previous studies (Mentioned in Table 4) on our dataset. Results obtained by this experiment on our test data are presented in Table 6. This is to note that we also presented the results of available studies with reference to the size of their datasets and discussed those in reference to our dataset in Table 5. From Table 6, we observe that almost all the standard ML models trained for four-class classification yielded an accuracy and F1 score under 0.60, an AUC-ROC between 0.70 and 0.80, and an AUPRC between 0.58 and 0.65. These results are indicative of a suboptimal performance (Table 6). This suboptimal performance could be because there is a high overlap between the feature distribution of successive severity classes. For example, the features of asymptomatic and mild, those of mild and moderate, and features of moderate and severe have overlapping distributions. So far, this has been the experience of medical doctors as well. This led to the inability of models to learn a proper classification boundary. Our approach of hierarchical classification tries to alleviate this problem by classifying the samples in a hierarchical/staged manner, due to which we could achieve accuracy and weighed an F1 score of 0.83, an AUC-ROC of 0.95, and an area under the precision-recall curve (AUPRC) of 0.91 on the test set. These results show a gain of 15-25% in all metrics compared to the other models in Table 6.

**Table 6:** Comparative performance of the CoSP model on four-class COVID severity classification with reference to the standard ML models. CoSP: COVID-19 Severity Predictor, SVM: Support Vector Machines, KNN: K-Nearest Neighbour, AUC-ROC: Area Under the Receiver Operating Characteristic Curve; AUPRC: Area Under the Precision-Recall Curve

Methodology	AUC-ROC	AUPRC	Accuracy	Weighted F1-Score	Previous Works
Neural Network	0.78	0.64	0.5	0.52	[14-15]
Random Forest	0.80	0.65	0.57	0.57	[12,13,15,22]
SVM	0.78	0.64	0.51	0.56	[11,14,16,22]
AdaBoost	0.76	0.58	0.61	0.57	[12,15]
Gradient Boosting Classifier	0.77	0.64	0.62	0.6	[12,14,15]
Bagging Classifier	0.71	0.58	0.51	0.55	-
KNN	0.73	0.60	0.61	0.58	[14]
CoSP (Ours)	0.95	0.91	0.83	0.83	

**Figure 9:**
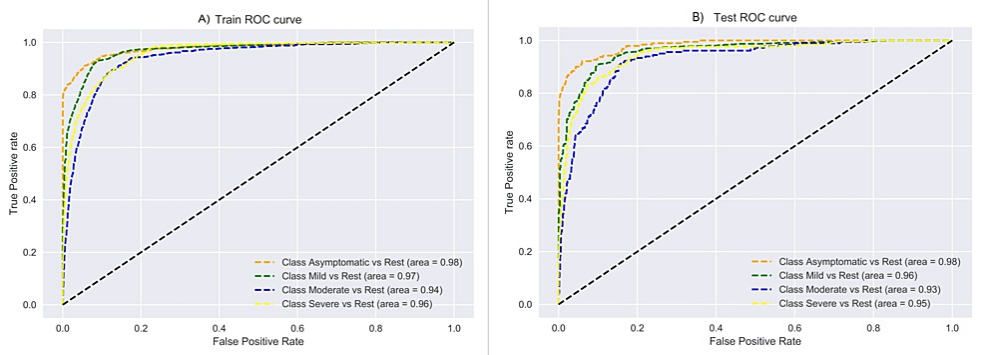
AUC-ROC performance curves of the model on (A) training and (B) testing data. The total data of 7,416 subjects were split randomly to 80% training data and 20% test data. AUC-ROC: Area Under the Receiver Operating Characteristic Curve

Further, to ascertain the appropriateness of the CoSP model, we studied the AUC-ROC performance curves on the train and test data, as shown in Figure 9. A good AUC-ROC value above 0.93 for all the individual classes, as well as the consistency in the train and test performance curves, demonstrate that the CoSP model has been trained well and that each class has been learned correctly. F1 score and AUPRC are better performance metrics for an unbalanced dataset as compared to the accuracy. These metrics focus on the positive class, which is of high importance in health care since only a relatively small fraction of the positive class population exists. Our CoSP model outperformed every other method, as shown in Table 6 w.r.t. weighted F1 score and AUPRC metrics. This result is much better than a random guess of 0.25 in a four-class classification problem and, hence, shows that CoSP performed satisfactorily.

Table 7 presents the performance of the trained CoSP model on the imputed and non-imputed subgroups of train and test datasets, and Table 8 on the subgroups of COVID-19 Wave 1 and Wave 2. For a four-class classification, the random guess performance is around 25%. Since the CoSP model achieved an AUC-ROC above 0.90, a weighted F1 score above or close to 0.80, and an AUPRC close to or above 0.90 in all sub-groups, it performed much better than a random guess. This shows that the model was trained properly. Further, from this table, we observe that the performance of the trained CoSP model on the subgroups of imputed and non-imputed, and COVID-19 Wave-1 and Wave-2 are comparable in the train and test datasets, as well as across groups. This is indicative that the trained model does not overfit, and its performance is not impacted by imputation. The consistency of results in COVID-19 Wave-1 and Wave-2 sub-groups also proves the generalizability of the CoSP model.

**Table 7:** Quantitative results of the CoSP Model on the imputed and non-imputed subgroups of the train and test datasets. AUC-ROC: Area Under the Receiver Operating Characteristic Curve; AUPRC: Area Under the Precision-Recall Curve

Dataset Subgroup	AUC-ROC	AUPRC	Weighted F1 Score
Total Train Data	0.96	0.91	0.85
Imputed Train Data (55.20% of Total Train Data)	0.96	0.91	0.87
Non-imputed Train Data (44.80% of Total Train Data)	0.93	0.88	0.78
Total Test Data	0.95	0.91	0.83
Imputed Test Data (54.16% of Total Test Data)	0.95	0.9	0.84
Non-imputed Test Data (45.84% of Total Test Data)	0.93	0.87	0.78

**Table 8:** Quantitative results of the CoSP Model on the COVID-19 Wave-1 and Wave-2 samples of the train and test datasets. AUC-ROC: Area Under the Receiver Operating Characteristic Curve; AUPRC: Area Under the Precision-Recall Curve.

Dataset Subgroup	AUC-ROC	AUPRC	Weighted F1 Score
Wave-1 Samples of Train Data (75.80% of Total Train Data)	0.95	0.91	0.86
Wave-2 Samples of Train Data (24.20% of Total Train Data)	0.95	0.91	0.82
Wave-1 Samples of Test Data (76.29% of Total Test)	0.94	0.9	0.82
Wave-2 Samples of Test Data (23.71% of Total Test)	0.96	0.91	0.85

We performed yet another experiment to assess the generalizability of the CoSP model. Here, the model was trained on the data of Wave-1, i.e., collected for the patients admitted before April 15, 2021, and the test set was formed by considering the most recent 20% data samples of Wave-2 collected for the patients admitted on or after April 15, 2021. This test set contained only 16 asymptomatic patients. Results are presented below in Table 9. From this table, we observe that, although the model was trained entirely on the Wave-1 data, it performed far better than the random guess of 25% on the Wave-2 test data on accuracy, F1-score, and AUPRC. The performance declined on the weighted F1-score. This decline in performance is understandable because different virus strains were involved in Wave-1 and Wave-2. Despite the decline in performance, the model still yielded an AUPRC of 0.83. This shows that the model is generalizable and shows that the results are not inflated in the existing proposed strategy. On the other hand, when we used a part of the Wave-2 data in the training, the model’s performance was even better, as shown in Tables 7 and 8. Both these results demonstrate that the proposed model strategy is robust and generalizable. Because the F1 score is calculated directly using precision and recall for each class, whereas the AUC-ROC is calculated using true-positive and false-positive rates in a one-versus-all fashion, we observe that F1 metric values are relatively lower than the AUC-ROC metric. Similarly, the AUPRC is a measure of a model’s predictive performance, which is based on the relationship between the positive predictive value (PPV) for the outcome and the model’s sensitivity for detecting patients’ severity. It is a crucial metric for assessing the precision and recall rate for an unbalanced dataset. A relatively low decline in an AUPRC in this experiment demonstrates the generalizability of the model.

**Table 9:** Performance of the CoSP model trained on data of patients admitted before April 15, 2021, and tested on data of patients admitted after April 15, 2021. AUC-ROC: Area Under the Receiver Operating Characteristic Curve; AUPRC: Area Under the Precision-Recall Curve

Dataset Split	AUC-ROC	AUPRC	Weighted F1 Score
Train (80%)	0.98	0.92	0.92
Test (20%)	0.86	0.83	0.70

Interpretation of model results

In order to assess the clinical significance of our results, SHAP analysis was done to observe the impact of individual features on risk stages predicted by glucocorticoid receptors (GCRS). Using SHAP analysis, the top few features were identified and checked with the literature for their significance with respect to COVID-19 severity. SHAP is basically a game theoretic approach that is used to interpret the decisions of a machine learning model. It helps in the interpretation of a trained machine learning model, identifying the relative importance of features leading to a prediction on a sample. This ‘SHAP’ package is available in Python.

**Figure 10:**
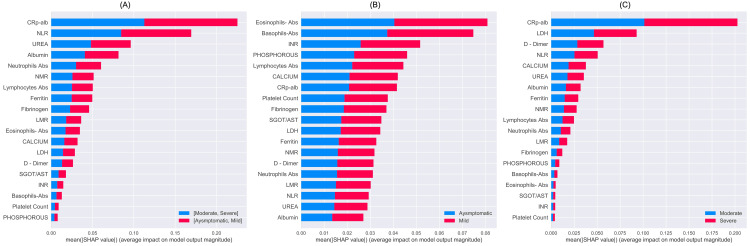
Mean SHAP values obtained from the trained classifier indicating feature importance. (A): SHAP results of classifier A, (B): SHAP results of classifier B, and (C): SHAP results of classifier C. A higher mean value indicates a higher importance of a feature in classifying the samples.

**Figure 11:**
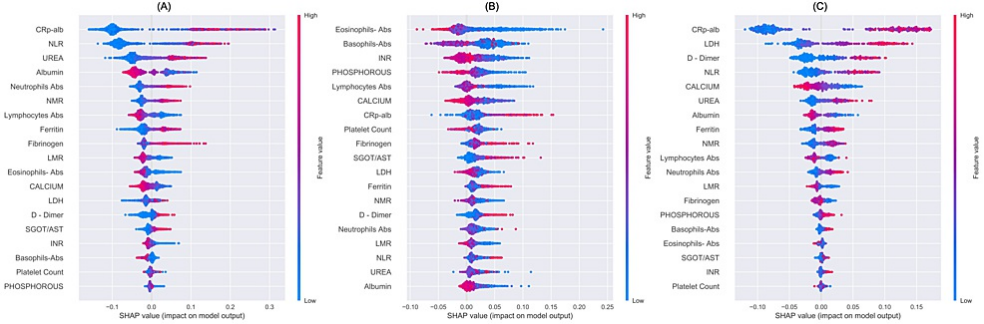
Model interpretation using SHAP (SHapley Additive exPlanations) summary plots. (A): SHAP results of classifier A, (B): SHAP results of classifier B, and (C): SHAP results of classifier C. These figures show the relative impact of different features (top to bottom) contributing to the classification. The topmost feature in each subfigure indicates a relatively highest relevance in classification. A positive SHAP value indicates contribution of a feature to class ‘1’, while a negative value indicates contribution to class ‘0’. Red color indicates a higher feature value, while a blue color indicates a lower feature value. For example, subfigure (c) indicates that a higher value of LDH contributes to severity class, while its lower value contributes to mild COVID-19 disease.

For Classifier A, which classifies the data into two groups of (asymptomatic, mild) and (moderate, severe), the following 19 features were found to be significant in the decreasing order of importance: CAR, NLR, urea, albumin, ANC, NMR, ALC, ferritin, fibrinogen, LMR, AEC, calcium, LDH, D-dimer, SGOT/AST, INR, ABC, platelet count, and phosphorus (Figure 10A). COVID-19 patients suffering from moderate or severe disease had raised CAR, NLR, urea, ANC, NMR, ferritin, fibrinogen, LDH, D-dimer, SGOT/AST, INR phosphorus and lower albumin, calcium, ALC, LMR, AEC, ABC, and platelet count levels (Figure 11A).

For Classifier B, which classifies the data into severity classes of asymptomatic and mild, the following features were found to be significant in the decreasing order of importance: AEC, ABC, INR, phosphorous, ALC, calcium, CAR, platelet count, fibrinogen, SGOT/AST, LDH, ferritin, NMR, D-dimer, ANC, LMR, NLR, urea, and albumin (Figure 10B). In comparison to asymptomatic patients, patients with mild disease had eosinopenia, basopenia, lymphopenia, hypocalcemia, hypophosphatemia, hypoalbuminemia thrombocytopenia, low INR, LDH, NMR, LMR levels and elevated CAR, SGOT/AST, fibrinogen, ferritin, urea, D-dimer, ANC, and NLR (Figure 11B).

For Classifier C, which classifies the data into severity classes of moderate and severe, the following features were found to be significant in the decreasing order of importance: CAR, LDH, D-dimer, NLR, calcium, urea, albumin, ferritin, NMR, ALC, ANC, LMR, fibrinogen, phosphorus, ABC, AEC, SGOT/AST, INR, and platelet count (see Figure 10C). Patients with severe COVID-19 showed higher CAR, LDH, D-dimer, NLR, urea, ferritin, neutrophilia, phosphorus and NMR, SGOT/AST, INR levels as well as hypocalcemia, hypoalbuminemia, lymphopenia, eosinopenia, thrombocytopenia and low levels of LMR, fibrinogen as compared to moderate disease (Figure 11C).

Development of a calculator for the assessment of disease severity

A user-friendly online calculator for the severity risk assessment of COVID-19 patients has been built based on 19 significant laboratory parameters (http://covidseverity.sbilab.iiitd.edu.in/). Django (https://www.djangoproject.com/), which is a Python-based web framework, was used to create this calculator. This framework allowed us to easily load and run the trained model, followed by passing the predicted output to the HTML5/CSS and JavaScript front end of the web app using asynchronous AJAX POST calls.

## Discussion

Severe acute respiratory syndrome coronavirus 2 (SARS-COV-2) is the third highly virulent coronavirus to emerge in the 21st century, following severe acute respiratory syndrome (SARS) in 2003 and Middle East respiratory disease (MERS) in 2012. It has put a huge strain on countries’ financial and medical resources because of its highly contagious nature, high mortality rate, and insufficient information regarding the virus. Patients with a severe COVID-19 infection are more likely to expire than those with a mild illness. The present challenge is to identify COVID-19 severity-specific biomarkers that might aid in the upfront identification of patients likely to develop severe disease in order to triage patients in resource-constrained settings of a pandemic.

A number of recent papers have been published on the use of ML for the task of severity prediction in COVID-19 research. For example, Yao et al. [11] adopted both urine and blood test-associated features and trained an SVM classifier for COVID-19 severity class versus non-severity class prediction. A total of 75 severe and 62 non-severe patients were used for training the model in this work. The samples were randomly split into 80% as training and 20% as test datasets in a stratified fashion. Laatifi et al. [12] combined biological and non-biological data from 194 severe and 146 non-severe patients with COVID-19 in Morocco and performed a binary classification on the dataset using features engineered by a common method of Uniform Manifold Approximation and Projection (UMAP). Navlakha et al. [13] created RF models for the severity classification of the COVID-19 disease into three categories: 71 severe-early (the patient required high levels of oxygen support within three days of being tested positive for COVID-19), 71 severe-late (the patient required high levels of oxygen after three days), and 206 non-severe (the patient never required oxygen support). The model was trained using 10-fold stratified cross-validation with a split of 90% train and 10% test for training each fold classifier. Aktar et al. [14] combined statistical and correlation methods with a number of ML algorithms to classify patients in either of the two categories as to whether to shift to the intensive care unit (ICU class) or shift to the normal ward (non-ICU class), out of which 264 had sufficiently severe symptoms to be admitted to the ICU. The model was trained using 80% of the data, and the rest was used for validation. In another work, Statsenko et al. [15] also classified patients into ICU versus non-ICU categories, with a number of 72 and 488 subjects, respectively, in each category. The model is trained using 10-fold cross-validation. Zhao et al. [16] developed a model for the prediction of severity in patients with moderate COVID-19 disease. A two-class SVM classifier was trained on a dataset of 112 patients in the mild class and 60 in the severe class by using fivefold cross-validation. Along similar lines, Huang [17] developed a model to predict severity discrimination among a dataset of 93 patients with mild symptoms and 32 patients with severe symptoms using a logistic regression (LR) classifier. Authors in [18-21] used LR ML models to classify patients into severe and non-severe groups. Five ML approaches - least absolute shrinkage and selection operator (LASSO) regression, logistic regression (LR), decision tree (DT), RF, and SVM, were employed for predicting severity versus non-severity in COVID-19 by Gong et al. in [22]. de Terwangne et al. [23] developed a Bayesian network analysis-based ML approach for predicting moderate, severe, and critical classes. Liang et al. [24] developed a risk score model. The study used LASSO regression, wherein 19 out of 72 variables were identified as important predictors and used in an LR model to build a risk score prediction model for severe vs. non-severe classes using a cohort of 1590 training patients and 710 test patients.

ML can be beneficial since it improves the functionality of conventional COVID-19 severity, mortality, and forecasting prediction techniques [3]. Based on the literature review of existing ML-based COVID-19 diagnostic methods [11-24], we observed a few limitations. The first concern was the use of a small sample size in most of these studies, which limits the ability to have reproducible results on a larger cohort or prospective patients. Additionally, a few studies have not used any imputation method to fill up the missing values in the dataset in a robust fashion, as it enables us to utilize the entire or almost entire data collected, helping us build models with large data sizes [14]. Furthermore, most of the studies, except [15], lack in handling class imbalance problems, which is one of the important ways to avoid overfitting the trained model. Another major drawback in all the studies is the consideration of only two classes, i.e., severe or non-severe class, while training a classifier, whereas COVID-19 is better managed clinically by identifying four classes/levels as asymptomatic, mild, moderate, and severe. Furthermore, many studies lack an interpretability/explainability analysis of the ML model that, if implemented, helps in understanding disease and its diagnostics and, hence, helps in gaining doctors’ trust in the ML models [11,13]. 

Therefore, this study had two objectives: 1) to develop a complete AI workflow for COVID-19 disease severity prediction at four disease levels using the features captured at the time of hospital admission, which will help in identifying high-risk patients for their prompt management; and 2) to identify crucial laboratory features via an interpretability analysis that can help clinician avoid an over-prescription of laboratory investigation, eventually leading to a reduced financial burden to patients and public-funded hospitals.

To this end, we developed and validated an AI-based complete workflow along with an online calculator for COVID-19 severity risk prediction using a routine baseline laboratory. The AdaBoost, gradient boosting, bagging, RF, SVM, KNN, NN models, and hierarchical approach were compared and evaluated. The hierarchical approach had the best performance in our study with an AUC-ROC of 0.95, an AUPRC of 0.91, and a weighted F1 score of 0.83. Few studies evaluating the application of ML techniques in diagnosis, as well as predicting the severity and mortality of COVID-19, have been published recently (Table 4). Our model suggested that the CRP to albumin ratio (CAR) is the most important determinant of COVID-19 severity. The CAR is a marker of systemic inflammation that has been found to be a significant severity predictor even in the present study. Karokoyun et al. also concluded that the CAR was a significant marker in early differentiation of severity in hospitalized COVID-19 patients who have longer hospital stays and higher mortality [30,31]. Other laboratory features identified to be significant for predicting severe disease in our study included NLR, NMR, ALC, LMR, ANC, AEC, ABC, phosphorus, SGOT/AST, albumin, urea, calcium, LDH, ferritin, platelet count, INR, fibrinogen, and D-dimer [32-34].

From the results of Table 5, we observed that, in the case of a two-way classification problem, the model of Laatifi et al. [12] achieved the highest AUC-ROC score of 1.0. However, it has certain limitations: 1) the dataset was small, consisting of data from only 337 subjects; 2) cross-validation was not carried out, which is required on a small dataset to ensure proper training of the model; and 3) uniform manifold approximation and projection (UMAP) feature reduction was carried out that leads to loss of interpretability of the trained model on the identification of class-specific features of the trained model. Next, the methods of Huang et al. [17] and Han et al. [20] yielded AUC-ROC scores of 0.94 and 0.98, respectively. These datasets are even smaller than that of the previous study and consist of only 47 and 125 subjects’ data, respectively. Thus, these predictors could be biased to the data seen and may not yield similar performance on the prospective subjects’ data. We also observe from Table 5 that, as the sample size increased, the AUC-ROC scores decreased. This shows that, indeed, small sample sizes may provide over-estimated performance. Only one study reported the weighted F1 score [14] and achieved a score of 0.94. Since the dataset is small, these results may not be generalizable to a larger dataset. In comparison to all these studies, the proposed CoSP-Classifier-A was built on 7,416 subjects’ data and, hence, is more generalizable compared to the other methods. CoSP-Classifier-A achieved an AUC-ROC score of 0.91, an accuracy of 86.65%, and a weighted F1 score of 0.87, which are higher than that on a reasonably sized dataset of, for example, Liang et al. [24] having 2,300 subjects’ data.

From Table 5, we also note that there is considerable heterogeneity across different studies. In order to validate any ML/DL model, there are generally two strategies: 1) either we have a publicly available dataset on which multiple authors have presented their results that allows us to benchmark the results of our model against their results, OR 2) we have different ML/DL models that researchers have used on datasets of this problem statement, and hence, these methods can be applied on our dataset. On this problem statement, we could not find one big available dataset on which researchers have benchmarked the results of their methods and, hence, could not benchmark our model on any such dataset. However, we did the literature review and noted the standard ML models used in the literature on different datasets. Hence, we applied these methods to our dataset and presented those results in Table 6. At the same time, we have presented the results of available studies concerning the size of their datasets and discussed them in reference to our dataset and results separately in Table 5, highlighting what could be the cause of good or not-so-good results reported by different studies on their datasets. Table 5 and its following discussion highlight the fact that working on medical datasets is often a challenge owing to the unavailability of large amounts of data, class imbalance in the datasets, difficulty in choosing an appropriate model, difficulty in applying ML correctly, and difficulty in inferring the results. Hence, we have also reported the interpretability of the trained model (later in this Section) via the identification of features contributing to the prediction of a class. A correct prediction of moderately severe/severe class helps the hospital administration maneuver resources and aids doctors in prescribing suitable treatment to save the most needy and critical patients.

SARS-CoV-2 infection affects multiorgan by intricate interaction of the immune, inflammatory, and coagulation pathways. It elicits hypoxia and hyper-activation of the immune system, which in turn activates neutrophils and monocytes-macrophage cells, leading to the exaggerated release of pro-inflammatory cytokine (CRP, ferritin, LDH), resulting in a cytokine storm [35]. Elevated CRP ≥ 130 mg/L and LDH > 246 U/L have been identified as the risk factors for severity, as well as mortality because they indicate inflammatory process activity [12,14,15,24,33,34]. Multiorgan failure, subsequent infection, and eventually death are all consequences of the cytokine storm. SARS-CoV-2 spike protein also mimics hepcidin, resulting in elevated ferritin levels, ferroptosis, and the formation of free toxic heme [35]. Lymphocytic apoptosis or atrophy of lymphoid organs and bone marrow cells is also caused by direct viral damage or cytokine storm, resulting in anemia, lymphopenia, eosinopenia, basopenia, and monocytopenia [30,34,35]. Other hematological parameters, such as neutrophilia, a high NLR, NMR, PLR, and low LMR, and platelet count, have also been identified as predictors of mortality and severe illness [11,12,14,24,33,34]. In the present study, NLR, ANC, NMR, ALC, LMR, AEC, ABC, and platelet count were found to be significantly deranged in patients with disease severity. We did not find an association of any of the RBC parameters (Hb, HCT, RBC, MCV, MCH, MCHC, RDW), TLC, AMC, PLR, and SII with disease severity in our cohort of COVID-19 patients.

Pulmonary microthrombosis and disseminated intravascular coagulation (DIC) are the most reported complications of COVID-19 due to endothelial damage, thrombo-inflammation, activation of platelets, increased blood flow stasis and viscosity, promoted thrombosis, inhibition fibrinolysis, impaired coagulation factors production due to liver damage, neutrophil extracellular trap (NET) formation, activation of complement systems [36]. COVID-19 patients with raised INR, D-dimer, and fibrinogen levels are associated with severe disease and worse prognosis [14,15,24,34,36,37]. In the present study, the prospective indicators for COVID-19 severity were raised D-dimer, fibrinogen, INR levels, and lower platelet counts. Therefore, recent studies, including the present study, justify the inclusion of coagulation studies at admission as crucial indicators of the severity of COVID-19, as well as the starting of anticoagulant therapy at the earliest.

Microemboli in the hepatic and renal vessels result in dysfunction of the liver and kidney, leading to electrolyte imbalance, as well as deranged liver and renal function tests [32]. The renin-angiotensin-aldosterone system (RAAS) is also dysregulated, resulting in an increase in angiotensin II, which leads to vasoconstriction, high blood pressure, increased vascular permeability, and electrolyte imbalance [32]. RAAS activation via angiotensin-converting enzyme-2 receptors increases urea reabsorption in renal tubules, resulting in hyper-uricemia. Several studies have found that elevated blood urea nitrogen (BUN), creatinine, hyperuricemia, bilirubinemia, hypoalbuminemia, elevated liver enzymes, A/G ratio, and electrolyte imbalance are predictors of severe COVID-19 disease and mortality [33,34]. However, in the present study, only uremia, hypoalbuminemia, hypocalcemia, raised SGOT/AST, and hyperphosphatemia were found to be associated with severe COVID-19.

It has been established that deranged laboratory parameters can increase the likelihood of severe COVID-19 disease and its eventual outcome. Determination of the most critical and significant variables can be used in the report constraints setting and can enable rapid clinical decision-making at the bedside. We evaluated a substantial sample size over a two-year period. We found the most significant 19 laboratory parameters and developed a readily available online calculator for risk stratification and prediction, which can be beneficial for clinicians in managing COVID-19 patients by accurately triaging high-risk patients at the earliest. Hence, the design of such a valid predictive model can not only improve the quality of care but can also improve the overall survival of patients [37]. Identification of potential biomarkers, which are useful predictors of disease severity and mortality, is very important. However, in order to utilize them in clinical practice, a simple, readily available model is required. With this work, we provide one such calculator, namely, the CoSP online calculator, and would like to recommend the researchers to build such online readily available resource calculators that can make research useful and translational to clinical practice.

One of the limitations of the present study is that it was a retrospective study design that led to a, despite large, imbalanced dataset. We utilized ML methods carefully in the CoSP model to handle class imbalance problems using class weighting while learning the decision boundaries during the training of the proposed model. Another limitation was that it was a single-center study, which may limit the generalizability of our model to the data collected from patients of different ethnicities and geographical locations around the globe. Another issue encountered was that the inflammatory (ferritin, LDH, CRP, procalcitonin, IL-6, CRP-ALB), coagulation (APTT, D-dimer, fibrinogen, PT, INR) and glycemic (Glu-R and HbA1c) features were missing in a significant number of patients because the pathophysiology of SARS-CoV-2 virus and clinical relevance of these features was not known in the initial phase of the pandemic. In general, missing data are a major problem in medical data and limit the usability of clinical data in real-life settings outside of clinical trials. It also limits the development of robust predictors. We overcame this limitation with a careful utilization of ML along with DL techniques to build a predictor. This design methodology is superior to the traditional ways of using ML because, as shown in this study, we could handle missing data efficiently through Datawig and could design a predictor that demonstrated good performance on quantitative metrics and reported relevant important features for the COVID-19 severity classes. We have also shown the performance of the developed CoSP model on different data subgroups: imputed and non-imputed subgroups of train and test datasets and also on the subgroups of COVID-19 Wave 1 and Wave 2. We have shown that the developed model performed much better than any random guess. We also performed another experiment in which the CoSP model was trained on the data of Wave-1 and tested on the data of Wave-2. Again, the trained model performed far better than the random guess. Both these results demonstrate that the proposed model strategy is robust and generalizable. Nevertheless, for a future study, we would like to do further in-depth analysis on patients diagnosed with COVID-19 in different waves to understand how the severity determinants vary due to different variants of the coronavirus. This would be possible with data being collected by AIIMS and would be made available for analysis.

## Conclusions

Identification of potential biomarkers that can predict COVID-19 disease severity can be of great utility for healthcare professionals to efficiently triage patients, personalize treatment, monitor clinical progress, and allocate proper resources at all levels of care to mitigate morbidity and mortality. We developed a robust and user-friendly ML model-based calculator for severity prediction (http://covidseverity.sbilab.iiitd.edu.in/) with high accuracy for confirmed COVID-19 hospitalized patients. With this calculator, patients with a risk of developing severe disease can be identified, enabling appropriate evaluation and optimal utilization of healthcare resources. We suggest that these laboratory features should be evaluated as part of baseline investigation at admission, and the capture of the rest of the features may be restricted to reduce an overall financial burden.

## Appendices

Random Forest Classifier

A random forest classifier is a model that fits a number of decision tree classifiers on sub-samples of the dataset with replacement. Sub-samples of the dataset are created using a process called bagging (bootstrap aggregation). Class label for a new test example is identified using majority voting of multiple trained decision trees. This also helps control overfitting.

Support Vector Machine

Support vector machines are a supervised machine learning algorithm that is mainly used for a classification task whose objective is to find a hyperplane in *N*-dimensional space (*N* = the number of features) that separates the classes. It transforms a training dataset into this higher dimensional space, followed by optimizing a hyperplane that separates the classes with minimum classification error. Here, support vectors are a set of data points that are closer to the hyperplane and influence the position and orientation of the hyperplane, eventually helping in the classification task.

AdaBoost Classifier

An AdaBoost classifier, which stands for adaptive boosting, is a machine learning algorithm that begins by fitting a classifier (a decision tree in our case) on the original dataset, followed by ensembling to combine the results from multiple trained classifiers using boosting. It builds these classifiers sequentially. Each successive classifier increasingly weighs the incorrectly classified instances so as to focus more on such difficult cases. Performance is boosted by the re-weighting of instances in accordance with the results of the previous classifier. 

Gradient Boosting Classifier

Gradient boosting classifier is an ensemble machine learning model that builds an additive model in a forward stage-wise fashion. It sequentially builds a set of trees. In each iteration, a tree is improved on the basis of its performance in the previous iteration. It comprises three elements: a loss function, a weak learner (e.g., a DT), and an additive model. It allows for the optimization of arbitrary differentiable loss functions. In each stage, N (N = the number of features) regression trees fit on the negative gradient of the binomial or multinomial deviance loss function.

Bagging Classifier

A bagging classifier is an ensemble-based machine learning algorithm that fits base classifiers (decision trees) each on random subsets of the original dataset and then aggregates their individual predictions (either by voting or by averaging) to form a final prediction. Such a meta-estimator can typically be used as a way to reduce the variance of a black-box estimator (e.g., a decision tree) by introducing randomization into its construction procedure and then making an ensemble out of it, which as a result, prevents overfitting.

K-Nearest Neighbor Classifier

K-nearest neighbor is a supervised machine learning algorithm that assumes that similar things exist in close proximity. It assigns by voting a datapoint its label by looking at its K-nearest neighbors in the original data using predefined Euclidean distance metrics.

Neural Network

Neural networks are a set of machine learning algorithms whose functioning mimics the working of a human brain. There is a set of interconnected neurons with some parameter values through which data are passed in a forward fashion to output some value. These values are compared with the expected output to calculate a loss value. This loss value is then used to update the parameters of the neural network in such a way that it minimizes the loss function using a process known as backpropagation. Thus, it is a supervised training mechanism. 

Accuracy 

It is the ratio of the correctly labeled samples to all the samples.

\begin{equation*} Accuracy = \frac{TP+TN}{TP+FP+TN+FN} \end{equation*} *Precision*

It is the ratio of the samples labeled correctly to the positive class by a model to all the samples labeled positive by that model.

\begin{equation*} Precision = \frac{TP}{TP+FP} \end{equation*}

Recall

It is the ratio of the samples labeled correctly to the positive class by a model to all the samples that actually belong to the positive class.

\begin{equation*} Recall (Sensitivity) = \frac{TP}{TP+FN} \end{equation*}

Specificity

It is the ratio of the samples labeled correctly to the negative class by a model to all the samples that actually belong to the negative class.

\begin{equation*} Specificity = \frac{TN}{TN+FP} \end{equation*}

*AUC-ROC* *Score*

It is calculated by calculating the area under the curve of the receiver operating characteristic curve created by plotting the true positive rates (TPR/Recall/Sensitivity) on the y-axis vs false positive rates (FPR) on the x-axis at various probability thresholds.

\begin{equation*} FPR = 1 - Specificity \end{equation*}

Weighted F1 Score

It is another metric used to calculate a classifier's performance. In an imbalanced multiclass scenario, the F1 scores for each class are calculated, and a class-weighted sum is done to get the final F1 score. These class weights are calculated using the class sample ratios in the dataset.

\begin{equation*} F1 = \frac{2 \times Precision \times Recall}{Precision + Recall} \end{equation*}
